# Lamin A/C regulates cerebellar granule cell maturation

**DOI:** 10.1007/s10565-025-10011-z

**Published:** 2025-04-05

**Authors:** Laura Vilardo, Ingrid Cifola, Marta Nardella, Paride Pelucchi, Maria Teresa Ciotti, Andrea Bianchi, Arianna Rinaldi, Ivan Arisi, Rossella Brandi, Mara d’Onofrio, Nicola Galvanetto, Giuliana Gatti, Myriam Catalano, Chiara Lanzuolo, Loredana Guglielmi, Igea D’Agnano

**Affiliations:** 1 CNR, Institute for Biomedical Technologies (ITB), Segrate, MI Italy; 2 Institute of Biochemistry and Cell Biology (IBBC), CNR, Monterotondo Scalo, RM Italy; 3 Istituto Nazionale Genetica Molecolare (INGM), Milan, Italy; 4 Department of Physiology and Pharmacology, Sapienza University, Rome, Italy; 5 European Brain Research Institute (EBRI) “Rita Levi Montalcini”, Rome, Italy; 6 University of Zurich, Zurich, Switzerland; 7 Department of Biotechnology and Translational Medicine, University of Milan, Milan, Italy; 8 Faculty of Health and Medical Sciences, School of Biosciences, University of Surrey, Guildford, UK

**Keywords:** Lamin A/C; Neuronal development; Cerebellar granule cells; Glutamate, Neurotoxicity

## Abstract

Lamin A/C is a nuclear type V intermediate filament protein part of the meshwork structure underlying the inner nuclear membrane (nuclear lamina), which plays numerous roles, including maintenance of nuclear shape, heterochromatin organization, and transcriptional regulation. Our group has demonstrated the role of Lamin A/C in different pathophysiological conditions. Here, we investigated for the first time how Lamin A/C affects neuronal maturation in rat cerebellar granule cells (GCs). Primary rat cerebellar GCs where we silenced the *Lmna* gene constituted our key model; this provided a rather homogeneous cellular system showing a neuronal population in vitro. We then validated our findings in another in vivo murine model with knock-out of the *Lmna* gene and in an in vitro human neuronal model with silencing of the *LMNA* gene. We observed across three different models that Lamin A/C down-regulation affects neurons maturation by protecting the cells from glutamate-evoked excitotoxicity and correlates with an inhibition of calcium influxes and a down-regulation of pro-inflammatory cytokine pathways. Consistent with previous findings from our group, this study corroborates that Lamin A/C plays a key role in neural development and opens new significant implications for a better comprehension of the mechanisms involved in neurodegenerative diseases, where changes in the nuclear envelope are linked to neuroinflammatory processes and damage.

**Supplementary Information:**

The online version contains supplementary material available at 10.1007/s10565-025-10011-z.

## Introduction

Nuclear lamins are nuclear type V intermediate filament proteins that create the meshwork structure underneath the inner nuclear membrane, the nuclear lamina (Turgay et al. 2017). Nuclear lamins were originally characterized as lamins A, B, and C (Gerace and Blobel 1980). Lamins A and C, and two variants known as AΔ10 and sperm-specific C2, belong to A-type lamins and are produced by the *LMNA* gene through alternative splicing. Three different B-type lamins are encoded by two other genes (B1 by *LMNB1* and *LMNB2*, and sperm-specific B3 by *LMNB2*) (Machiels et al. 1996; Höger et al. 1990; Furukawa and Hotta 1993). The nuclear lamina together with other nuclear envelope proteins is involved in numerous roles, including maintenance of nuclear shape and structure, assembly and disassembly of the nucleus, heterochromatin organization, transcriptional regulation, and other nuclear functions (Burke and Stewart 2013; Zuela et al. 2012; Simon and Wilson 2013). It is well established that mutations affecting lamins and lamina-associated proteins are the leading cause of various human diseases called “laminopathies,” such as cardiac and muscular dystrophy, lipodystrophy, and premature aging disorder (Schreiber and Kennedy 2013). In physiological conditions, B-type lamins are expressed in most or all cell types, whereas A-type lamins are not expressed in immature cells during the early developmental stages in mice and appear late in embryonic development (Burke and Stewart 2013; Zuela et al. 2012; Röber et al. 1989), being only detected in differentiated cells (Röber et al. 1989; Constantinescu et al. 2006; Zhang et al. 2013; Yang et al. 2019) Lamin A/C role in differentiation is mediated by its mechanosensing function, as it can sense the cell’s external environment and can differentiate accordingly, with a stiff environment increasing osteogenesis and a soft environment increasing adipogenesis (Yang et al. 2019; Swift et al. 2013). In the brain, the crucial role of B-type lamins in its development has been extensively characterized (Vergnes et al. 2004; Coffinier et al. 2010), while the role of Lamin A/C has not been fully elucidated yet. A-type lamins have a peculiar pattern of expression in the adult brain, being Lamin C rather than Lamin A preferentially expressed (Zuela et al. 2012). This is very likely due to microRNA-mediated (miR-9) removal of prelamin A transcripts. (Jung et al. 2012).

As down-regulation of Lamin A/C is important for neuroblastoma development in vivo by increasing tumour cell stemness (Maresca et al. 2012; Nardella et al. 2015; Guglielmi et al. 2017), we hypothesised that these proteins may play an important role in neuron development and maturation under physiological conditions. Primary cultures of post-mitotic granule neurons from postnatal rat cerebellum represent an exceptional model for molecular and cellular biological studies of neuronal development and function (Borodinsky et al. 2015). Cerebellar granule cells (GCs) are the smallest and most abundant neurons in the entire mammalian central nervous system and are located within the internal granular layer, just below a monolayer of Purkinje cells in the cerebellum (Consalez et al. 2021). As part of the maturation of postnatal cerebellum, they undergo a well-defined differentiation program characterized by sequential and progressive maturation stages, where the final phase is the development of dendrites and synaptic connections with excitatory mossy fibres and inhibitory GABAergic terminals from Golgi type II neurons (Consalez et al. 2021). Excitatory neurotransmitters, such as glutamate, actively promote the maturation and differentiation of GCs (Consalez et al. 2021). Here, we used rat cerebellar GCs and this well-defined maturation process via glutamate stimulus to investigate the role of A-type lamins in the maturation of cerebellar GCs under physiological conditions.

## Materials and methods

### Primary cell cultures of rat cerebellar granule cells

For each isolation of cells, mixed-sex litters of 10 Wistar 8-day-old rat pups were obtained. The pups were euthanized by decapitation on the day of experiment, without prior use of anaesthesia. Animals were kept in standard cages under a 12-h light–dark schedule at a constant temperature of 21 °C with food and water ad libitum, in an authorized facility at Santa Lucia Foundation, Rome (Italy). All procedures involving rats complied with the Istituto Superiore di Sanità (Italian Ministry of Health) and current European Ethical Committee guidelines (directive 86/609/ECC). Efforts were made to minimize animal suffering and to reduce the number of animals used. Primary cultures enriched in post-mitotic cerebellar granule cells (GCs) were obtained by dissociation of cerebella from 8-day-old rat pups, following the procedure described by Levi et al. (1984). Cells were seeded (3 × 10^6^ cells/dish) in 35-mm plastic dishes previously coated with poly-L-lysine using Gibco Basal Medium Eagle (BME, ThermoFisher Scientific, Walthman, MA, USA) supplemented with 10% heat-inactivated foetal calf serum (Gibco, ThermoFisher Scientific), 2 mM glutamine (Gibco, ThermoFisher Scientific), and 25 mM KCl and 100 µg/ml gentamycin (Gibco, ThermoFisher Scientific). To ensure a good purity of cerebellar granule cells 24 h after cell plating, we added Ara-C (10 mM; Sigma Aldrich, St. Louis, MO, USA) to the culture medium to prevent proliferation of non-neuronal cells. This protocol typically yields cultures of cerebellar granules that are highly enriched (> 90%) and extremely pure since Ara-C functions as anti-mitotic agent by inhibiting DNA synthesis in dividing cells (Drejer and Schousboe 1989). This mechanism ensures that all non-neuronal, dividing cells, potentially present in our cultures, are negatively selected and washed away by protecting neuronal cells that are less proliferating.

### SH-SY5Y cell cultures

In this study, we also used two SH-SY5Y clones, well characterized in previous papers (Maresca et al. 2012; Nardella et al. 2015), derived from the parental SH-SY5Y human neuroblastoma cell line (ATCC), *LMNA*-knockdown (KD) and Mock cells that were obtained and cultured as previously described (Maresca et al. 2012). Cells were cultured in a 1:1 mixture of Eagle’s Minimum Essential Medium and F12 medium (Gibco, ThermoFisher Scientific) supplemented with 10% Fetal Bovine Serum (FBS; Hyclone, ThermoFisher Scientific), 2 mM L-glutamine, 0.5% non-essential amino acids, 0.5% sodium pyruvate and 1% penicillin and streptomycin, in the presence of blasticidin.

### Lentiviral infection for generation of *Lmna*-KD rat cerebellar GCs

GCs dissociated from rat cerebella were seeded (3.5 × 10^5^ cells/dish) in 35-mm plastic dishes previously coated with poly-L-lysine. After four hours, they were exposed to the virus-containing supernatant from pLenti6/V5-GW-miR-*LMNA* or pLenti6/V5-GW-miRNeg (Maresca et al. 2012). After 18 h, fresh medium was added. The miRNA expression was monitored by checking the  *Lmna* gene expression.

### *Lmna* knock-out (KO) mice

The generation of Lamin Δ8–11 knock-out mouse (*Lmna* − / −) used in this study has been already described (Sullivan et al 1999; Jahn et al. 2012; Cesarini et al. 2015). The animals were kept, as described above, in an authorised facility at Santa Lucia Foundation, Rome (Italy). Cerebella of 8-day-old wild type (n = 8) and *Lmna*-KO (n = 9) mice were dissociated, and cultures enriched in granule neurons were obtained according to the procedure described by Levi et al. (1984) as above described.

### Glutamate excitotoxicity

The evaluation of glutamate excitotoxic effect was performed in all the three models employed using multiple cell viability assays.

For rat cerebellar GCs, after 2, 5 and 8 days in culture, they were washed once in Locke solution (in mM: 154 NaCl, 5.6 KCl, 3.6 NaHCO3, 2.3 CaC12, 1.0 MgC12, 5.6 glucose, 10 HEPES, pH 7.4) and exposed to a 100 µM glutamate pulse in Mg^2+^ -free Locke solution for 30 min at room temperature. Cells were subsequently washed in Mg^2+^ -free Locke solution and returned to the incubator in their original medium. After 18 h, counting of the numbers of intact nuclei was used to determine the number of viable cells, as reported by Soto and Sonnenchein (1985) and modified for counting cerebellar granule cells following the procedure described by Volonté et al. (1994).

The same assay was performed in *Lmna*-KO mouse cerebellar GCs after 8 days in culture. The cells were treated as described above for rat GCs.

For human SH-SY5Y cells, Mock and *LMNA*-KD cell cultures were exposed to different concentrations of glutamate (0.1, 10 and 60 mM) for 24 h. Then, cells were harvested, washed once in PBS and analysed for viability by using propidium iodide (PI) and FACS. Cells were suspended in a solution of PBS containing 10 µg/ml PI and incubated for 1 min at room temperature in the dark, then directly measured by a FACSCalibur cytometer (Becton Dickinson) and CellQuest Pro BD software (Becton Dickinson). Flow cytometry data were analyzed by FlowJo^TM^ data analysis platform v.8.0 (Becton Dickinson). PI cell viability assay was also used to evaluate cell viability in SH-SY5Y Mock and *LMNA*-KD cells in the presence of the Ca^2+^ chelator 1,2-bis(o-Aminophenoxy)ethane-N,N,Nʹ,Nʹ-tetraacetic Acid Tetra(acetoxymethyl)Ester (1.5 mM; BAPTA-AM, Molecular Probes). Cells were pre-incubated 20 min with the chelator, which was maintained during the treatment in the presence of glutamate 60 mM for 24 h. The PI assay was performed as described above and samples analyzed by FACS. The presence of viable cells after exposure of Mock and *LMNA*-KD SH-SY5Y to glutamate 60 mM in absence or presence of the Ca^2+^+ chelator was also evaluated by the Cell Counting Kit-8 (CCK8) assay (Merck KGaA, Darmstadt, Germany). 96-well plate cell cultures were exposed to glutamate 60 mM in the absence or presence of the Ca^2+^ chelator as described above. After 24 h the CCK-8 solution was added to each sample following the manufacturer’s protocol for 3 h at 37 °C. Absorbance was then measured at 450 nm using the Varioskan™ LUX Multimode Microplate Reader (Thermofisher Scientific, Waltham, MA, USA).

### Annexin V assay

Mock and *Lmna*-KD GCs were treated with 100 µM glutamate as described above. They were then harvested, pooled with the supernatant, washed once in PBS and processed for Annexin V assay. Vybrant Apoptosis assay kit (Invitrogen) was used following the manufacturer’s protocol and samples analyzed by FACSCalibur cytometer (Becton Dickinson) and CellQuest Pro BD software (Becton Dickinson). Flow cytometry data were analyzed by FlowJo™ data analysis platform v.8.0 (Becton Dickinson).

### Confocal Immunofluorescence of cerebellar tissue

Confocal analysis of Lamin A/C in foetal (E10) and neonatal (P10 and P18) rat cerebella were performed. After perfusion with saline, under deep anesthesia (60 mg/kg Nembutal i.p.), followed by cold 4% PFA in 0.1 M Phosphate Buffer, pH 7.4, the brains were excised, cerebella isolated and cryoprotected in 30% sucrose/PB at 4° C. They were then frozen with dry ice and cut into 40-μm transverse sections with a freezing microtome. Non-specific staining was blocked by incubating sections in blocking buffer (0.25% Triton X-100, 5% normal donkey serum in PBS) for 1 h at room temperature. Antibodies used: mouse monoclonal antibody anti-NeuN (A60, Sigma Aldrich) and goat polyclonal anti-Lamin A/C (N18, Santa Cruz Biotechnology, TX, USA). All antibodies were diluted in PBS containing 5% normal donkey serum and 0.01% Triton X-100. After overnight incubation and three washes in PBS, primary antibody staining was revealed using fluorescence-conjugated secondary antibodies (Thermo Fisher Scientific) at 2 µg/mL. Sections were washed three times in PBS, mounted on gelatin-coated slides, and coverslipped in ProLong Gold Antifade Reagent (Thermofisher Scientific). Nuclei were stained with DAPI (P36935, Invitrogen, Carlsbad, CA, USA). For the negative control, sections were treated following the previously outlined protocol, except for omitting the primary antibody. Sections were examined under a confocal laser scanning microscope (LeicaSP5, Leica Microsystems, Germany) under sequential mode to avoid crosstalk between channels. Image processing and final figures were done by using AdobePhotoshop 7 and Adobe Illustrator 10. Fluorescence intensity measurement was performed by ImageJ software. The Integrated Density value (the product of Area and Mean Gray Value) was calculated for each image (n = 3) of nuclei, NeuN and Lamin A/C. Quantification was performed by calculating the Ratio between the NeuN or Lamin A/C fluorescence and the nuclei fluorescence.

### Immunofluorescence of primary rat GCs

Rat GC neuronal cultures were washed twice with PBS and fixed in 4% (w/v) paraformaldehyde for 15 min at room temperature. Cells were permeabilized with 0.1% (v/v) Triton X-100/PBS, pH 7.4, for 4 min at room temperature. Coverslips were saturated with 2% BSA and 10% normal goat serum (NGS) for 3 h followed by incubation overnight at 4 °C in a humidified chamber with the mouse monoclonal antibody anti-NeuN (A60, Sigma Aldrich) and goat polyclonal anti-Lamin A/C (N18, Santa Cruz Biotechnology, TX, USA). Unbound antibody was removed by three washes with PBS and bound antibodies were detected by incubation with donkey anti-mouse Alexa 488 (Thermo Fisher Scientific) and donkey anti-goat Alexa 594 (Thermo Fisher Scientific) secondary antibodies at room temperature for 30 min. Nuclei were stained with nuclear dye 4,6-diamidino-2-phenylindole dihydrochloride (DAPI; Sigma, St. Louis, MO, USA) 1:1000 in PBS for 5 min. Controls were performed by omitting the primary antibody. Then, coverslips were mounted on Superfrost glass slides using the Prolong Gold Antifade Mounting (Thermo Fisher Scientific) and kept at −20^◦^C before image analysis. Immunofluorescence images were acquired with an epifluorescent microscope (Leica CTR5500; Leica Microsystems, Mannheim, Germany) equipped with a CCD camera (Leica). Final figures were assembled by using Adobe Photoshop 7 and Adobe Illustrator 10. Images are representative of at least three independent experiments. Fluorescence intensity measurement was performed by ImageJ software. The Integrated Density value (the product of Area and Mean Gray Value) was calculated for each image (n = 3) of nuclei, NeuN and Lamin A/C. Quantification was performed by calculating the Ratio between the NeuN or Lamin A/C fluorescence and the nuclei fluorescence.

### Western blotting

Cultured cells were washed twice with 1X PBS and then incubated for 1 min in 1X PBS added with 0.5 mM phenylmethylsulphonyl fluoride (PMSF; Sigma-Aldrich) and 1X Complete Protease Inhibitors (Sigma), then scraped, harvested, and briefly sonicated. Proteins were suspended in urea buffer (8 M urea, 100 mM NaH2PO4, and 10 mMTris pH 8) and the protein concentration was calculated with Bradford assay (Bio-Rad Laboratories S.r.l., Segrate, Italy). Thirty µg of proteins were subjected to SDS–polyacrylamide gel electrophoresis with NuPAGE kit (Life Technologies, Carlsbad, CA, USA) according to manufacturer's instructions. Resolved proteins were blotted overnight onto nitrocellulose membranes, which then were blocked in 1X PBS containing 5% non-fat milk for at least 1 h. Blots were incubated with the following primary antibodies: goat polyclonal anti-Lamin A/C (N-18; Santa Cruz Biotechnology, Dallas, TX, USA); mouse monoclonal anti-GAPDH (6C5; Merck Life Science, Milan, Italy). After four washes (10 min/each) in 1X PBS and 0.1% Tween20, the membranes were incubated for 45 min with the appropriate secondary antibody: donkey anti-goat IRdye800 (LI-COR Biosciences, Lincoln, NE, USA) or donkey anti-mouse IRdye800 (LI-COR Biosciences). The membranes were then analysed with the Licor Odyssey Infrared Image System (LI-COR Biosciences) in the 800 nm channel. Densitometry analyses were performed using ImageJ software.

### Intracellular calcium analysis

Mock and *Lmna*-KD rat GCs were cultured in 96-well plates as described above. After 8 days in vitro (8DIV), GCs were washed once in Locke solution (in mM: 154 NaCl, 5.6 KCl, 3.6 NaHCO3, 2.3 CaC12, 1.0 MgC12, 5.6 glucose, 10 HEPES, pH 7.4) and exposed to a 100 µM glutamate pulse in Mg^2+^-free Locke solution for 30 min at room temperature. Cells were subsequently washed in Mg^2+^-free Locke solution and incubated with Ca^2+^ sensor Fluo-4 (Fluo-4 Direct Calcium Assay Kits, Molecular Probes, Eugene, OR, USA) for 40 min at 37 °C in medium containing probenecid 5 mM following manufacturer’s protocol. Green fluorescence (excitation at 494 nm and emission at 516 nm) was then measured using the Wallac 1420 Victor2 fluorescence microplate reader (Perkin Elmer, Shelton, Connecticut, USA).

Mock and *LMNA*-KD SH-SY5Y cells were cultured on Poly-D-Lysine coated glass culture slides at a confluence of about 70%. Cells were then incubated with Ca^2+^ sensor Fluo-4 (Fluo-4 Direct Calcium Assay Kits, Molecular Probes) for 40 min at 37 °C in medium containing probenecid 5 mM following manufacturer’s protocol. Cells on the slide were then transferred to the stage of an upright fluorescence microscope (Bx51, Olympus, Tokyo, Japan) and continually superfused with an extracellular medium (Locke solution, see above). After 90 s, glutamate was superfused, within the same extracellular medium, at 60 mM concentration (IC_50_). Fluo-4 was excited by light at 490 nm from collimated LED (Thorlabs, Newton, NJ, USA), for 100 ms every cycle of image acquisition (1 s). LED emission was filtered through interference band–pass filters centered on peak wavelength, attenuated with a neutral density filter and conveyed onto the sample by reflection off a DM480HQ dichromatic mirror (Olympus). Fluorescence emission was collected through an interference filter (BA495–540HQ, Olympus) using a water immersion objective (20x, N.A. 0.95, XLumPlanFI, Olympus). Images were formed on a CCD camera (PCO Sensicam, 100 ms exposure time/frame) controlled by software developed in the laboratory. Images were analyzed with software developed in the laboratory using the MATLAB platform (Release 14, MathWorks, Inc., Natick, MA, USA). Fluo-4 traces were generated by averaging pixel signals within regions of interest (ROIs) corresponding to individual cells.

Intracellular calcium was also analysed by flow cytometry. Mock and *LMNA*-KD SH-SY5Y cells were cultured in 35-mm Petri dishes at a confluence of about 70%. Cells were then exposed to Ca^2+^ sensor Fluo-4 (Fluo-4 Direct Calcium Assay Kits, Molecular Probes) for 40 min at 37 °C in medium containing probenecid 5 mM following manufacturer’s protocol. Cells were collected in trypsin/EDTA solution, centrifuged at 200 × *g* for 5 min and suspended in Locke solution. Glutamate 60 mM was then added to the cell suspension for 30 min at room temperature and the cell-associated fluorescence was measured by a FACSCalibur cytometer (Becton Dickinson) and CellQuest Pro BD software (Becton Dickinson).

### RNA extraction

Total RNA was isolated from the different cell types using the Total RNA purification kit (Norgen Biotek, Thorold, ON, Canada), according to manufacturer’s protocol. Total RNA was quantified by Quant-it™ RiboGreen RNA Assay Kit (Invitrogen, ThermoFisher Scientific) according to the manufacturer’s instructions. The absorbance of the samples was measured by a multimode microplate reader (Varioskan Lux, Life Technologies) at a wavelength of 530 nm. RNA integrity was determined with a BioAnalyzer 2100 instrument, using a RNA 6000 Nano kit (Agilent Technologies, Santa Clara, CA, USA). Samples with an RNA Integrity Number (RIN) index lower than 8.0 were discarded.

### Microarray gene expression profiling of rat GCs

Gene expression profiling of *Lmna*-KD and Mock rat cerebellar GCs was performed using the Agilent Whole Rat Genome 4 × 44 K microarray platform (Agilent Technologies). Briefly, the cyanine 3-CTP labeled cRNA samples were prepared using the Agilent Low Input Linear Amplification Kit (Agilent Technologies). After hybridization and washing, array images were acquired using the Agilent Scanner G2564C (Agilent Technologies) and signals were extracted by Agilent Feature Extraction software (ver 10.7.3.1), according to the standard Agilent one-color gene expression extraction protocol (GE1_1100_Jul11). Data quality filtering and normalization were performed and differentially expressed genes (DEGs) in *Lmna*-KD vs. Mock cells were identified as those having a fold-change ratio |*Lmna*-KD/Mock|> 1.5 in linear scale. Probe annotation file was downloaded from NCBI GEO repository (Platform GPL7289, Last update date Apr 06, 2012). ClusterProfiler R package (v.3.18.1) (Yu et al. 2012) was used for the functional enrichment analysis of annotated DEGs and Gene Ontology (GO) Biological Process (BP) and Molecular Function (MF) categories were retrieved from the Rattus Norvegicus org.Rn.eg.db R package (v.3.12.0). The whole list of genes on the Agilent array (17,366 annotated genes) was used as background. A false-discovery-rate (FDR, Benjamini–Hochberg correction) threshold < 0.05 was applied to all the annotation terms to define statistically significant enrichments.

### RT-qPCR

The expression of differentiation and stemness genes in rat GCs was evaluated by RT-qPCR at different days during culturing.

To validate the microarray data obtained in *Lmna*-KD rat GCs, we also performed in *Lmna*-KO mice the RT-qPCR of some selected genes whose expression was up- or down-regulated by *Lmna* silencing in rat cerebellar GCs in vitro. Total RNA was isolated from wild type (n = 4) and KO (n = 4) mice as above described. RNA was quantified by Quant-it™ RiboGreen RNA Assay Kit (Invitrogen) according to the manufacturers’ guidelines. RNA integrity was checked on 2200 TapeStation System (Agilent Technologies). Samples with a RIN lower than 8.0 were discarded.

For all samples, RNA was reverse-transcribed using the High-Capacity cDNA Reverse Transcription Kit (Applied Biosystems, Waltham, MA, USA) according to the manufacturer’s instructions. Equal amounts of cDNA were then subjected to real-time qPCR analysis on QuantStudio™ 7 Flex Real-Time PCR System (Applied Biosystems) with PowerUp™ SYBR™ Green Master Mix (Applied Biosystems).

The primers listed below (r for rat, m for mouse) were used at final concentration of 200 nM using *Tbp* or *Ppia* as endogenous controls: *rTbp* (For: 5’-CCCACCAGCAGTTCAGTAGC-3’, Rev: 5’-CAATTCTGGGTTTGATCATTCTG-3’); *rLmna* (For: 5’-GAGCAAAGTGCGTGAGGAGT-3’, Rev: 5’- TCCCCCTCCTTCTTGGTATT-3’); *rProm1* (For: 5’-GCCCAAGCTGGAAGAATATG-3’, Rev: 5’- CAGCAGAAAGCAGACAATCAA-3’), *rNes* (For: 5’-TCCCTTAGTCTGGAAGTGGCTA-3’, Rev: 5’- GGTGTCTGCAAGCGAGAGTT-3’); *rZic2* (For: 5’-TCAACATACCAACCCATAGC-3’, Rev: 5’-AAAAATACATTCACAAGCGTTGG-3’); *rGabra6* (For: 5’-AATGTCAGTCGGATTCTTGACA-3’, Rev: 5’- TGTTTTGACTTCTGTTACAGCAC-3’); *mPpia* (For: 5’-CCCACCGTGTTCTTCGACAT −3’, Rev: 5’- CCAGTGCTCAGAGCTCGAAA-3’); *mCcl5* (For: 5’-CCAATCTTGCAGTCGTGTTTGT-3’, Rev: 5’-CCCTCTATCCTAGCTCATCTCCA-3’); *mCcl7* (For: 5’-TCACCAGTAGTCGGTGTCCC-3’, Rev: 5’-ACCCACTTCTGATGGGCTTC-3’); *mCxcl10* (For: 5’-CGTGTTGAGATCATTGCCACG-3’, Rev: 5’-TGGTCTTAGATTCCGGATTCAGA-3’); *mKcnma1* (For: 5’-CGTGGGTCTGTCCTTCCCTA-3’, Rev: 5’-TCCCAGGGTTAATTAATATTCGGCT-3’); *mKcnmb2* (For: 5’-GCAGAGCGTGTGGACAGAAG-3’, Rev: 5’-GGCAAGGGTACTGAGAGAGC-3’); *mKcnt1* (For: 5’-CCACCACTGGCTATGAGGAC-3’, Rev: 5’-AGGGTGTTCTGGTGATGATCG-3’).

### Microarray gene expression profiling of SH-SY5Y human neuronal cells

Gene expression profiling of *LMNA*-KD and Mock SH-SY5Y neuroblastoma cells was performed using the Agilent one-color microarray standard protocol and was already deposited in GEO Series GSE30677 (Maresca et al. 2012). Briefly, the cyanine 3-CTP labeled cRNA samples were prepared using the Agilent Low Input Linear Amplification Kit (Agilent Technologies) and hybridized onto Agilent 4 × 44 K Whole Human Genome oligonucleotide microarray (GEO platform GPL6480). Images were acquired using the Agilent Scanner G2564B by Agilent Feature Extraction software (ver 10.1), using the one-color gene expression extraction protocol (GE1_107_Sep09). DEGs were defined as those having a fold-change ratio |*LMNA*-KD/Mock|> 1.5 in linear scale. Probe annotations were downloaded from the Agilent portal web (https://earray.chem.agilent.com, annotation version 08-Nov-2022).

Functional enrichment analysis of annotated DEGs was performed using ClusterProfiler R package (v.3.18.1) (Yu et al. 2012) and Gene Ontology (GO) Biological Process (BP) and Molecular Function (MF) categories retrieved from the Homo sapiens org.Hs.eg.dbR package (v.3.12.0). The whole list of genes on the Agilent array (14,554 annotated genes) was used as background. A false-discovery-rate (FDR, Benjamini–Hochberg correction) threshold < 0.05 was applied to all the annotation terms to define statistically significant enrichments.

### Statistical analysis

Statistical analysis was performed using GraphPad Prism 5 software (GraphPad software, Inc., La Jolla, CA, USA). One-way ANOVA was used for groups of data. Unpaired student’s t test was used for comparison of pairs of data.

## Results

### Lamin A/C increases during rat cerebellar GC maturation

**Fig. 1:**
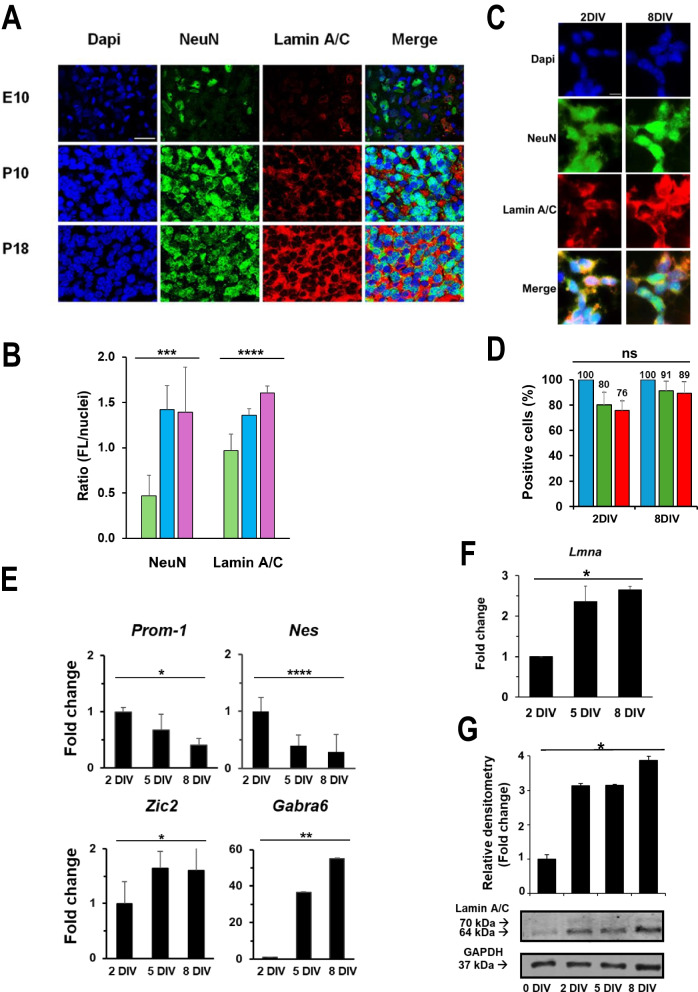
Lamin A/C increases during rat cerebellar GC maturation. **A** Representative confocal images of rat cerebellar slices of embryonic (E10) and post-natal (P10, P18) days immunostained for Lamin A/C (red), Neuronal Nuclear Antigen (NeuN, green) and nuclei (blue). Scale bar: 40 µm. **B** Quantification of the fluorescence in E18 (green), P10 (blue sky) and P18 (purple) images in Fig. 1A was performed by ImageJ software and expressed as the ratio between the intensity fluorescence of NeuN or Lamin A/C and the nuclei fluorescence. Three different microscopic fields were considered in each condition.** C** Representative confocal images of NeuN (green), Lamin A/C (red) and nuclei (Dapi, blue) in GCs at 2 and 8 days in vitro (DIV). Scale bar: 15 microns. **D** Counts of positive fluorescent cells were performed by ImageJ software in GCs at 2 and 8 DIV. Blue (nuclei), green (NeuN), red (Lamin A/C). Four different microscopic fields were considered in each condition. The number on each histogram represents the percentage of positive cells. **E** RT-qPCR analysis of the indicated genes in GCs at 2, 5 and 8 DIV. Data are reported as the level of mRNA relative to 2 DIV and are expressed as mean ± standard error (SE; n = 3). **F** RT-qPCR analysis of *Lmna* gene expression in GCs at 2, 5 and 8 DIV. Data are reported as the level of mRNA relative to 2 DIV and are expressed as mean ± SE (n = 3). **G** Representative blots of the Lamin A/C protein expression in GCs at 0, 2, 5 and 8 DIV and relative densitometric analysis. GAPDH was used as protein loading control (n = 3). All the experiments were repeated at least three times with independent results, the number (n) of experiments is shown in each case. One-way ANOVA test: *p ≤ 0.05; **p ≤ 0.01; ***p ≤ 0.001; ****p ≤ 0.0001

Since Lamin A/C has been proposed to be essential during neuronal differentiation, we analyzed its expression in rat cerebellum in vivo. Figure 1A shows representative immunohistochemistry confocal images in which Lamin A/C increased during cerebellum granule cells (GCs) development, an excellent experimental model for molecular and cell biological studies of neuronal development and neurodegenerative diseases (Amadoro et al. 2004; Amadoro et al. 2007; Barbato et al. 2010; Shedenkova et al. 2023).

During GCs development from embryonic stage (E10) to postnatal stages (P10, P18), Lamin A/C increment was also accompanied by the up-regulation of the well-known neuronal marker NeuN (Fig. 1A and B).

We then obtained primary cultures of rat cerebellar GCs (Volonté et al. 1994) from 2 up to 8 Days In Vitro (DIV). An immunofluorescence staining with the marker NeuN demonstrated a high purity of our GC cultures being expressed in almost all the cells in culture (80 and 91% of positive nuclei for NeuN at 2 and 8 DIV, respectively) (Fig. 1C and D). In vitro maturation of GCs from rat cerebellum was confirmed by the significant increment of expression of the differentiation-related genes *Zic2*, a zinc finger protein specifically expressed in the cerebellum during differentiation, and *Gabra6*, GABAA receptor alpha 6 subunit marking cerebellar GC maturation, and the subsequent significant decrement of the expression of the stemness-related genes *Prom-1*, coding for the CD-133 marker, and *Nes*, coding for nestin protein, an intermediate filament normally present in neural cells during development from 2 to 8 DIV (Fig. 1E). During this neuronal maturation time, we observed an increase in the expression of *Lmna* gene of about 2.5-fold from day 2 up to day 8 (Fig. 1F) and of Lamin A/C protein of about threefold at day 2 up to day 8 with respect to day 0 (Fig. 1G). These data suggest that Lamin A/C expression coincides with neuronal maturation in our model.

### *Lmna* knock-down prevents the complete maturation of GCs in vitro

**Fig. 2:**
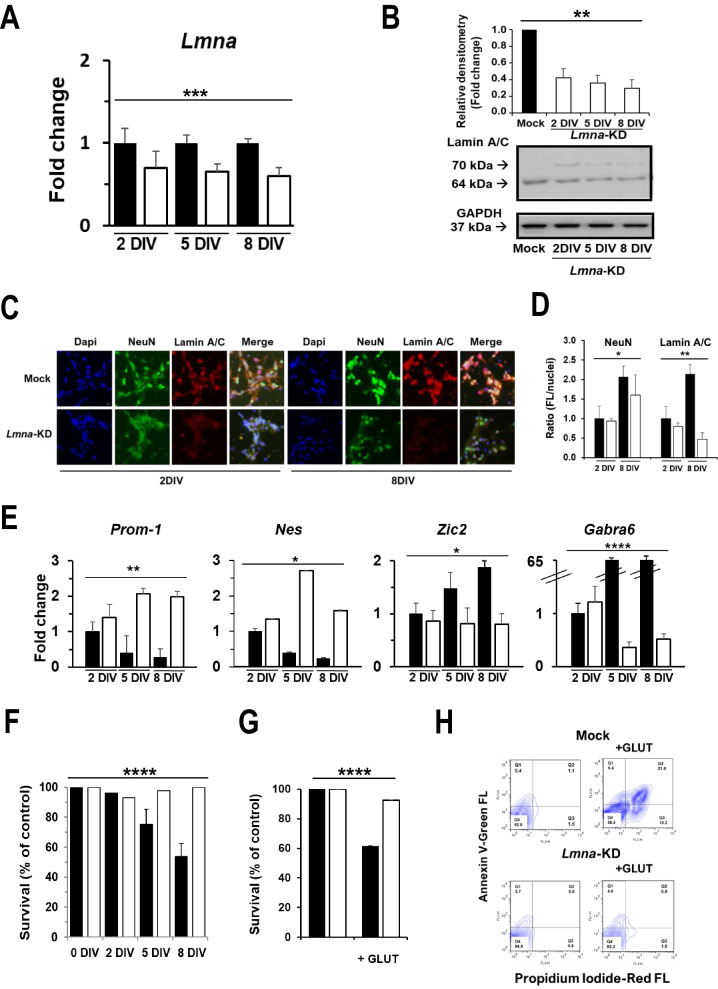
Lmna knock-down decreases glutamate sensitivity in rat GCs. **A** RT-qPCR analysis of *Lmna* mRNA levels in *Lmna*-KD rat GCs (white) relative to a sister control culture (black). Data are expressed as mean ± SE (n = 5). **B** Representative blots, and relative densitometric analysis, of Lamin A/C protein in control (Mock) and *Lmna*-KD GCs at 2, 5 and 8 DIV. GAPDH was used to control protein loading (n = 3). **C** Representative fluorescent images of NeuN (green), Lamin A/C (red) and Dapi (blue), in Mock and *Lmna*-KD GCs at 2 and 8 DIV. Scale bar:15 microns. **D** Quantification of the fluorescence in Mock (black) and *Lmna*-KD (white) GC images in Fig. 2C at 2 DIV and 8 DIV was performed by ImageJ software and expressed as the ratio between the intensity fluorescence of NeuN or Lamin A/C and the nuclei fluorescence. **E** RT-qPCR analysis of the indicated genes in *Lmna*-KD rat GCs (white). Data are reported as the fold change of the mRNA expression relative to Mock 2 DIV (black) and are expressed as mean ± SE (n = 5). **F** Cell viability analysis in Mock (black) and *Lmna*-KD (white) GCs exposed to a pulse of 100 µM glutamate for 30 min, as evaluated by counting intact nuclei (see Methods for details) at different DIV. Data represent the means ± SD (n = 5) of viable cells calculated as the ratio (%) between intact nuclei counted after the glutamate pulse and those counted in a sister control culture and are expressed as % of control. **G** Cell viability analysis in Mock (black) and *Lmna*-KD (white) GCs exposed to a pulse of 100 µM glutamate for 30 min, at 8 DIV as evaluated by CCK8 cell viability assay. Data represent the means ± SD (n = 3) of absorbance as measured by spectrofluorometer and are expressed as % of control. **H** Representative FACS cytograms showing cell death analysis after glutamate exposure by annexin V assay in Mock and *Lmna*-KD GCs at 8 DIV (n = 3). In the cytograms are reported the percentage of cells in each quadrant (Q). Q1 = PI positive non-viable cells that underwent necrosis; Q2 = PI + Annexin V positive late apoptotic cells; Q3 = Annexin positive early apoptotic cells; Q4 = negative viable cells. All the experiments were performed at least three times with independent results, the number (n) of experiments is shown in each case. When SD bar is not evident, it is included in the histogram. One-way ANOVA test: *p ≤ 0.05; **p ≤ 0.01; ***p < 0.001; ****p < 0.0001

To determine whether the increase in the levels of Lamin A/C protein observed from 2 to 8 DIV is necessary for the maturation of GCs in vitro, we inhibited its expression by silencing the *Lmna* gene in our rat GC model, using a lentiviral vector expressing a miRNA that directly targets *Lmna* mRNA (Maresca et al. 2012). The significant reduction in the expression of *Lmna* gene in GC primary cultures was evident already after 2 DIV (Fig. 2A); as well, western blotting analysis showed a reduced expression of the protein at 2, 5 and 8 days after *Lmna* silencing (Fig. 2B).We also analyzed Lamin A/C protein in Mock and KD GCs at single cell level using immunofluorescence microscopy. Figure 2C and D show a reduction of Lamin A/C level already after 2 days in vitro. The concomitant analysis of the NeuN neuronal marker showed a significant increase between 2 and 8 DIV, while a slight decreased expression level was observed between Mock and KD cells (Fig. 2C and D).

Coherently, the RT-qPCR analysis of the differentiation and stemness markers strongly suggested that the maturation of GCs does not occur in vitro in silenced samples, since the differentiation markers *Gabra6* and *Zic2* decreased, and the stemness (*Prom-1* and *Nes* markers) was maintained after *Lmna* silencing (Fig. 2E). The expression of *Prom-1* and *Nes* encoding proteins was also analysed by FACS and their expression levels showed the same trend as the gene expression (Supplementary Fig. 1).

Beyond the molecular effect on stemness and differentiation markers, we aimed to understand whether a reduction in the expression of Lamin A/C may have exerted an important functional effect on the maturation of GCs in vitro. We used the neurotransmitter glutamate to verify the maturation of rat GC primary cultures in conditions where Lamin A/C was physiologically expressed. We observed that as the maturation of GC primary cultures proceeds in vitro, they become gradually more and more vulnerable to the excitotoxic effect of glutamate, and after 8 days of in vitro culturing, cell viability significantly decreased (survival was 71.4 and 51.1% at 5 and 8 DIV, respectively) (Fig. 2F and G). Interestingly, when analysing cell survival after glutamate stimulation in *Lmna*-KD GCs, we found that neuronal cells do not respond to the excitotoxic insult of glutamate, being cell viability 90.7% at 5 DIV and 97.7% at 8 DIV (Fig. 2F and G). Annexin V assay performed at 8 DIV demonstrated that KD GCs respond less than Mock cells to excitotoxic glutamate in term of cell death (about 7% cell death in *Lmna*-KD CGs vs 45% in Mock GCs; Fig. 2H). Taken together, these data suggest that the decreased *Lmna* expression could prevent glutamate-mediated neural death. In other words, the presence of *Lmna* is necessary for GC primary cultures to remain responsive to glutamate during GC maturation.

### *Lmna* knock-down induces changes in the gene expression profile of rat GCs

To unravel genes and biological processes potentially linked to Lamin A/C function in the maturation of rat cerebellar GCs, we decided to take advantage of an “omic” approach and investigate the whole gene expression profile of *Lmna*-KD GCs as compared to Mock GCs.

Using the Agilent Whole Rat Genome microarray platform, we identified 141 up- and 108 down-regulated probes in *Lmna*-KD rat GCs vs Mock GCs, which after annotation resulted in 50 up- and 71 down-regulated rat known genes (Supplementary Table 1).

**Fig. 3:**
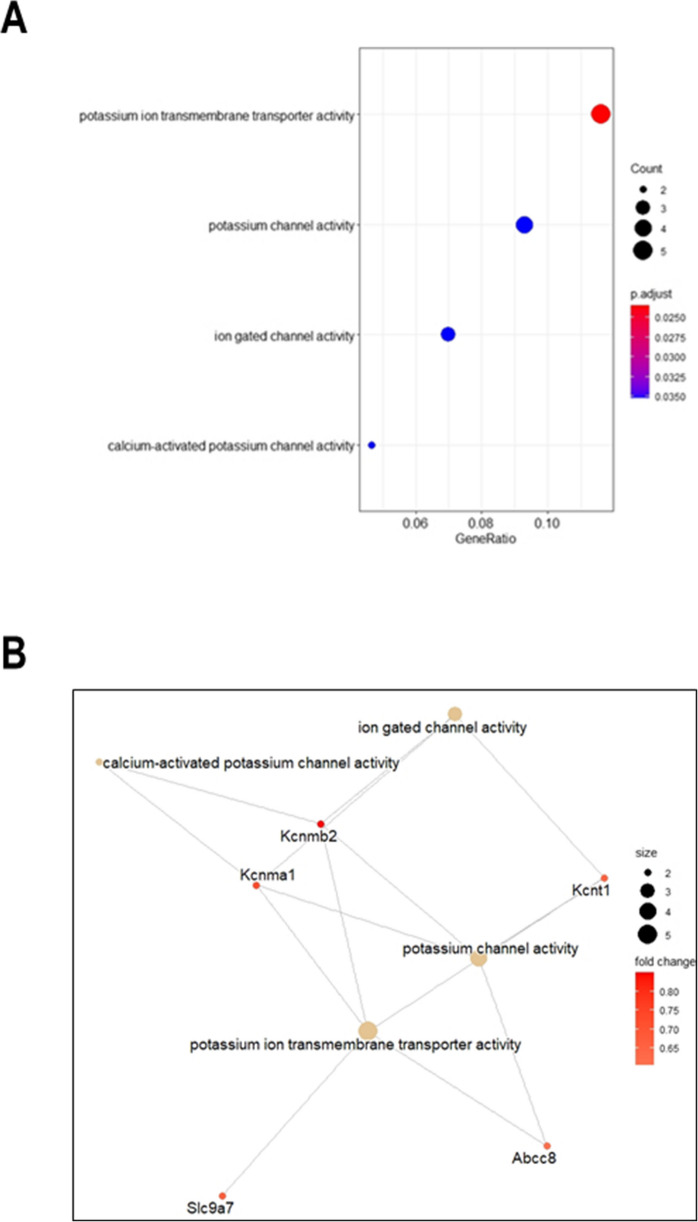
Functional enrichment analysis of up-regulated DEGs in *Lmna*-KD vs Mock rat GCs. **A** Bubble plot showing a selection of Gene Ontology Molecular Functions (GO-MF) terms enriched by up-regulated DEGs. Bubble size represents the number of DEGs involved in each function, while colour gradient indicates statistical significance of the enrichment (adjusted p-values). **B** Network plot of the selected GO-MF terms and corresponding up-regulated DEGs involved in each function. Circle size of the terms represents the number of DEGs involved, while colour gradient indicates fold-change values of DEGs

Functional enrichment analysis for Gene Ontology (GO) categories showed that genes up-regulated after *Lmna* silencing are mainly involved in potassium channel activity, important in regulating neuronal excitability and to prevent excitotoxic damage (Stocker 2004; Dolga et al. 2011) and include *Kcnma1*, *Kcnmb1*, *Kcnt1, Slc9a7*, and *Abcc8* genes (Fig. 3 A and B).

**Fig. 4:**
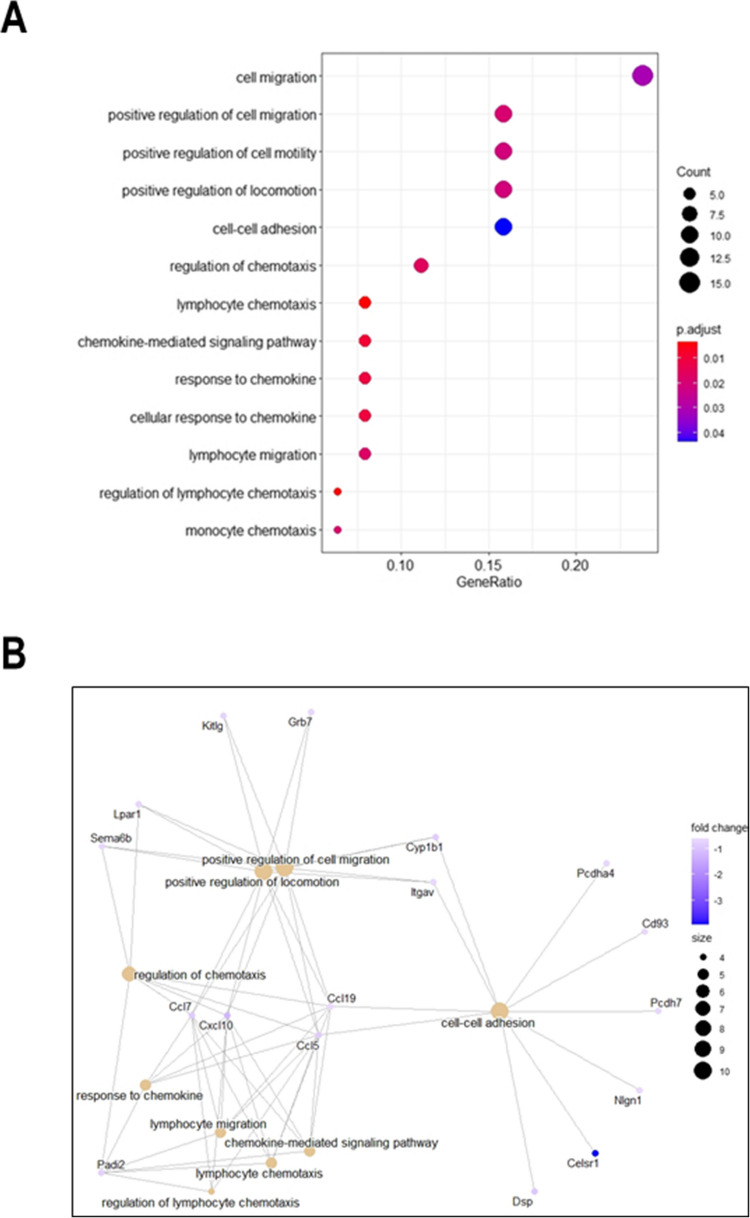
Functional enrichment analysis of down-regulated DEGs in *Lmna*-KD vs Mock rat GCs. **A** Bubble plot showing a selection of Gene Ontology Biological Process (GO-BP) terms enriched by down-regulated DEGs. Bubble size represents the number of DEGs involved in each process, while colour gradient indicates statistical significance of the enrichment (adjusted p-values). **B** Network plot of the selected GO-BP terms and corresponding down-regulated DEGs involved in each process. Circle size of the terms represents the number of DEGs involved, while colour gradient indicates fold-change values of DEGs

On the other hand, down-regulated genes enriched terms related to chemokine activity, chemotaxis, cell migration, cell motility and cell–cell adhesion, all activities essential for neuronal maturation, specifically with down-regulation of *Cxcl10*, *Ccl5*, *Ccl7* and *Ccl19* genes (Fig. 4 A and B). Taken as a whole, these findings support what we above observed after the down-regulation of Lamin A/C and glutamate stimulation in vitro. Particularly, they provide useful insights on the potential functional mechanisms of Lamin A/C in preventing GCs to respond to the glutamate stimulus.

### *Lmna* − / − mouse GCs show an increased survival upon glutamate and a change in gene expression

To corroborate the results observed with regard to the importance of Lamin A/C in the maturation of neuronal cells, we used a previously described *Lmna* − / − mouse model (Sullivan et al 1999; Jahn et al. 2012; Cesarini et al. 2015).

**Fig. 5:**
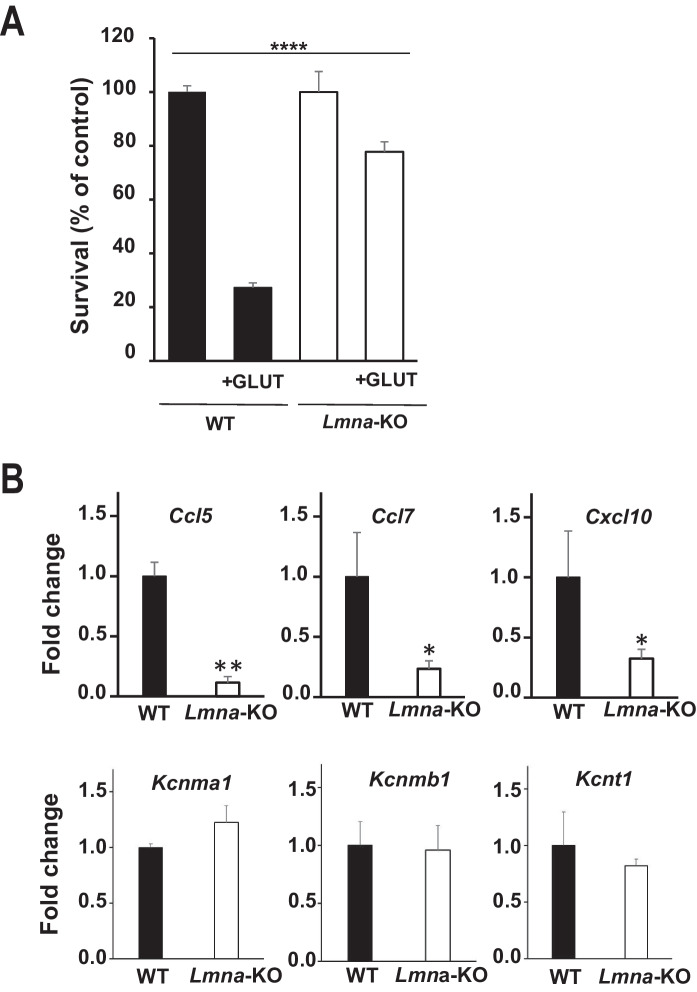
Effect of *Lmna*-KO on mouse GC glutamate sensitivity and gene expression. **A** Cell viability analysis in wild-type (WT, black) and *Lmna*-KO murine GCs (white) exposed to a pulse of 100 µM glutamate for 30 min (see Methods for details) at 8 DIV, as evaluated by counting intact nuclei. Data represent means ± SE (n = 5) of viable cells calculated as the ratio (%) between intact nuclei counted after the glutamate pulse and those counted in a sister control culture. When SE bar is not evident, it is included in the histogram. One-way ANOVA test: ****p < 0.0001. **B** RT-qPCR validation of rat GC gene expression profiling results. Fold-change values of the mRNA expression levels of *Ccl5*, *Ccl7*, *Cxcl10*, *Kcnma1*, *Kcnmb1* and *Kct1* in GCs derived from *Lmna*-KO (white) and WT (black) mouse brains are reported. Data represent means ± SE across ten independent mice (n = 10: 5 WT and 5 KO mice). Student's t-test: * p < 0.05; **p < 0.01

Cerebella from wild-type (WT) and *Lmna*-KO mice were isolated to obtain primary cultures of GCs. Following stimulus with glutamate, cerebellar GCs from *Lmna*-KO mice showed a significantly increased survival rate (about 80% survival), as compared to cerebellar GCs from WT mice (about 30% survival) (Fig. 5A).

Notably, we confirmed a substantial down-regulation of *Ccl5* (p < 0.01), *Ccl7* (p < 0.05) and *Cxcl10* (p < 0.05) gene expression in *Lmna*-KO GCs as compared to WT GCs (Fig. 5B). On the other hand, the up-regulation of potassium channels genes observed in vitro was less evident in vivo. In fact, *Lmna*-KO GCs showed a marginally increased expression of *Kcnma1* as compared to WT GCs, but this difference was not statistically significant, while we did not observe any remarkable variation in the gene expression levels of *Kcnmb1* or *Kcnt1* (Fig. 5B). Although the response of these cells to glutamate is comparable to that of the silenced cells, the pattern of gene expression is only partially superimposable, particularly for genes involved in migration and not for those related to potassium channel activity. This discrepancy is certainly due to the milieu in which the KO cells were grown as opposed to the silenced cells grown in a wild-type context. The data from the KO model support the importance of Lamin A/C in the maturation of cerebellar GCs.

### Intracellular calcium fluxes and glutamate-evoked excitotoxicity is modulated by *LMNA* expression

**Fig. 6:**
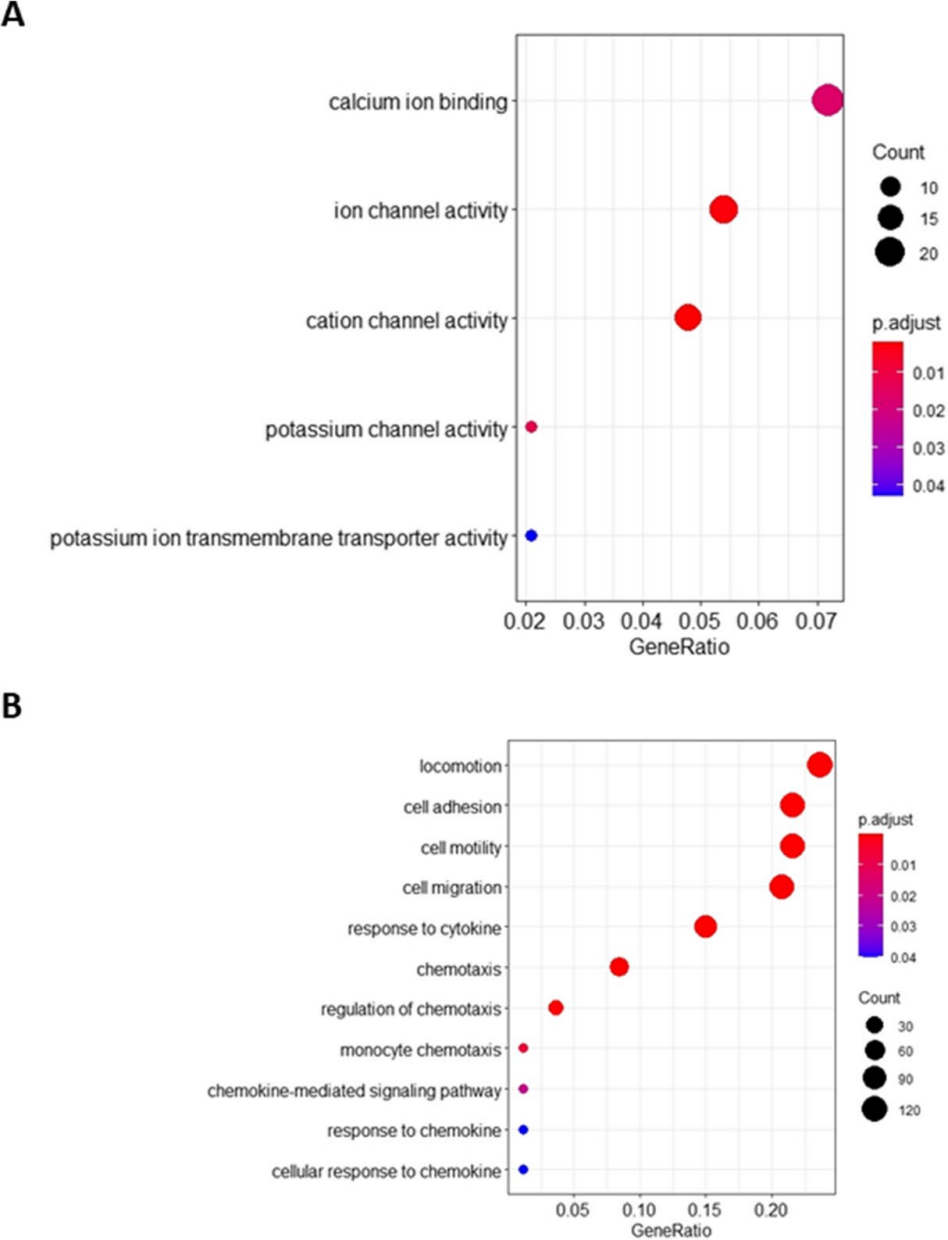
Functional enrichment analysis of DEGs found in *LMNA*-KD vs Mock SH-SY5Y human neuronal cells. **A** Bubble plot showing a selection of Gene Ontology Molecular Functions (GO-MF) terms enriched by up-regulated DEGs. **B** Bubble plot showing a selection of Gene Ontology Biological Process (GO-BP) terms enriched by down-regulated DEGs. Bubble size represents the number of DEGs involved in each term, while colour gradient indicates statistical significance of the enrichment (adjusted p-values)

Using SH-SY5Y cells as our model allowed us to better understand the mechanism by which glutamate-evoked excitotoxicity is modulated by *LMNA* expression, performing functional assays in a more controllable way and reducing variability, whilst minimizing the number of euthanized animals. In addition, this human model was already characterised in our laboratory to investigate the role of *LMNA* silencing in neuronal differentiation processes, stemness and tumorigenicity of neuroblastoma tumours (Maresca et al. 2012; Nardella et al. 2015). We first confirmed in SH-SY5Y cells the gene expression changes observed in rat GCs after *Lmna* silencing. We re-analysed the SH-SY5Y microarray raw data previously deposited in GEO repository (GSE30677), and we found a total of 404 up- and 590 down-regulated genes in *LMNA*-KD vs Mock SH-SY5Y cells. Functional enrichment analysis for GO categories confirmed the up-regulation of potassium channel activity (Fig. 6A) as well as the down-regulation of cytokine response, chemotaxis, cell migration, motility and adhesion after *LMNA* silencing (Fig. 6B), thus confirming the altered expression of the machinery required for neuronal maturation.

**Fig. 7:**
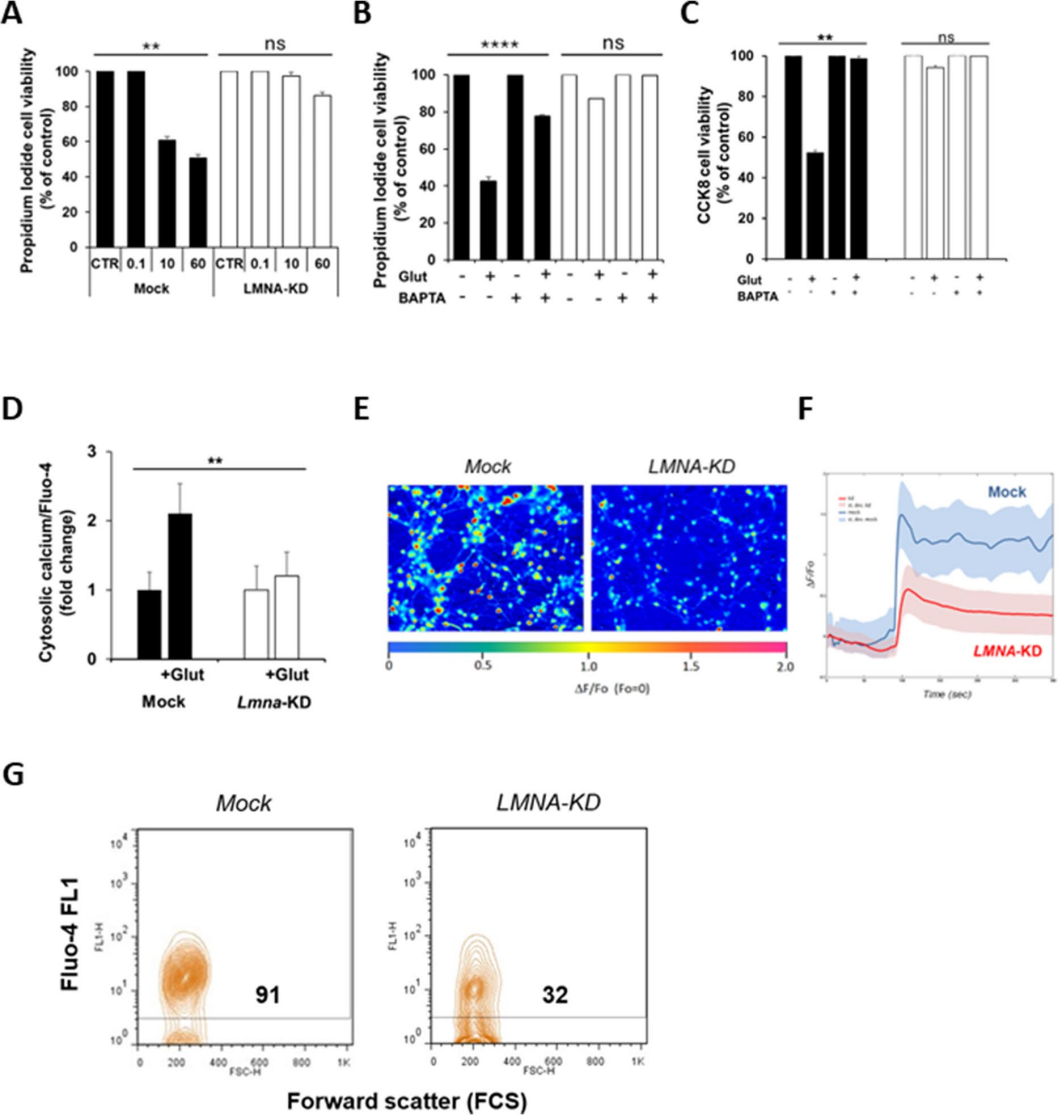
*LMNA* knock-down reduces intracellular calcium and prevents glutamate-evoked excitotoxicity in SH-SY5Y cells. **A** Cell viability analysis in Mock (black) and *LMNA*-KD (white) SH-SY5Y cells exposed at the indicated concentrations (in mM) of glutamate for 24 h, as evaluated by propidium iodide cell viability assay. **B** Cell viability analysis in Mock (black) and *LMNA*-KD (white) SH-SY5Y cells exposed at 60 mM of glutamate for 24 h, in presence or not of BAPTA calcium chelator, as evaluated by propidium iodide cell viability assay. **C** Cell viability analysis in Mock (black) and *LMNA*-KD (white) SH-SY5Y cells exposed at 60 mM of glutamate for 24 h, in presence or not of BAPTA calcium chelator, as evaluated by CCK8 cell viability assay. **D** Cytosolic calcium concentration in Mock (black) and *Lmna*-KD (white) GCs exposed to 100 µM of glutamate for 30 min, as evaluated by Fluo-4 Direct Calcium Assay Kits (Molecular Probes). **E** Representative confocal microscopic images which show the fluorescence intensity of intracellular calcium content in Mock and *LMNA*-KD SH-SY5Y cells, as evaluated by the Fluo-4 Direct Calcium Assay Kits (Molecular Probes) after glutamate exposure. **F** Time course of the change in fluorescence during the glutamate superinfusion in Mock and *LMNA*-KD SH-SY5Y cells. The dispersion around the graph lines represents the SD of the different measurements. ΔF/Fo = variation of fluorescence intensity divided by the fluorescence intensity at time zero. **G** Representative FACS cytograms of Fluo-4-FL1 cell-associated fluorescence in Mock and *LMNA*-KD SH-SY5Y cells, as evaluated by Fluo-4 Direct Calcium Assay Kits (Molecular Probes). The number in the cytogram represents the percentage of Fluo-4 positive cells in both lines after glutamate exposure. All experiments were performed at least three times with independent results. One-way ANOVA test: **p ≤ 0.01; ****p < 0.0001; ns = not significant. Glut, glutamate

In *LMNA*-KD cells, we also confirmed a significant increase in the survival of these cells when exposed to glutamate stimulus (Fig. 7A). Intracellular calcium is necessary to neuronal differentiation (Gallo et al. 1987; Franklin et al. 1995; Galli et al. 1995; Lampe et al. 1995). In fact, the glutamate-evoked excitotoxicity observed in the control cells was inhibited by about 50% when the cells were exposed to a calcium chelator during the treatment with glutamate (Fig. 7B and C), confirming that excitotoxicity in our experimental condition is calcium influx-dependent. We therefore hypothesised that intracellular calcium may mediate the Lamin A/C-dependent neuronal maturation process. For that reason, we looked at the levels of intracellular calcium in *Lmna*-KD GCs and in *LMNA*-KD SHSY5Y cells vs respective Mock cells using a fluorescent assay. *LMNA* knock-down significantly reduced intracellular calcium fluxes by 50% in the GCs (Fig. 7D) and more than 80% in SH-SY5Y cells (Fig. 7E and F). Intracellular calcium in *LMNA*-KD SH-SY5Y was also analysed by FACS confirming a reduction by about 70% with respect to Mock cells (Fig. 7G). Altogether these results confirm that Lamin A/C modulates calcium influx.

## Discussion

Lamin A/C has been the subject of interest of many groups across the globe since 1983. However, to the best of our knowledge, efforts have been mainly focused on finding mutations and aberrant variants of this protein rather than on the effects of the expression variations, particularly during development in physiological and pathophysiological conditions. This is one of the first studies focusing on a physiological model of neural maturation to investigate the role of Lamin A/C in this complex process. Here, we chose a model of rat cerebellar GCs that provides a rather homogeneous cellular system showing an enriched neuronal population in vitro (glia expansion is prevented by inhibiting mitosis), as we verified by the expression of the NeuN neuron differentiation marker, found expressed in our GC cultures in about 90% of cells. These cells preserve in culture the same mechanisms of glutamatergic fibres formation occurring during development in vivo such as neurotoxicity caused by excessive release of glutamate, considered for several years a key element of neuropathologies of both the acute and the chronic type (Choi 1988; Coyle and Puttfarcken 1993; Contestabile 2002; Magdaleno Roman and Chapa 2024).

We observed that Lamin A/C expression increases during the maturation of rat cerebellar GCs both in vivo and in vitro, hypothesizing its involvement in the maturation process. Taking advantage of Lamin A/C down-regulation by silencing the gene, we demonstrated that the protein expression positively correlates with the expression of neuronal differentiation markers and negatively with the expression of the stemness markers, PROM1 and its encoding protein CD133 as well as the neuroepithelial stem cell protein (Nestin), that are widely recognized as antigenic markers of stem cells (Bernal and Arranz 2018; Pleskač et al. 2024). Our data are in agreement with other previously published papers showing a crucial role of A-type lamins in the differentiation and stemness processes of other cell types (Nardella et al. 2015; Zhang et al. 2019; Jung et al. 2013). In fact, the *LMNA* gene plays a crucial role in regulating gene expression during development and in the initiation of differentiation. Its abnormal expression or mutation has been linked to various pathologies (Zaragoza et al. 2024; Perovanovic and Hoffman 2018; Talwar et al. 2014).

The presence of heterogeneity widely characterizes all the steps of neural development starting from progenitors and the advent of single-cell transcriptomics has allowed a fine classification of neurons and their progenitors, uncovering a previously underestimated heterogeneity in space and time (Alieh and Herrera, 2023;Agalliu and Schieren 2009; Li et al. 2019; Kempf et al. 2021). In addition, unlike established cell lines that provide unlimited supplies of homogeneous cells, primary cultures are characterized by heterogeneity. The preparation and culture of primary cells is much more challenging, and this is especially true for neuronal cells. Primary cell cultures are not immortal and hence the number of cells available for experiments is much more limited (Gordon et al. 2013; Piwocka et al. 2024; Harper 2024). For all these reasons, Lamin A/C expression is not expected to be homogeneous across immature and mature granule neurons, particularly in primary cultures. This was evidenced especially in the immunofluorescence images, showing the presence of variability in the expression of both Lamin A/C and NeuN.

Furthermore, we observed that the genetic ablation of Lamin A/C by *LMNA* knock-out or silencing changes the responsiveness of cerebellar GCs to the excitotoxic insult with glutamate, strongly suggesting the inhibition of GC maturation. Cerebellar GC neurons have been shown to proceed into their maturation process when cultured in vitro for 8 days. During their primary cultures, they become gradually more vulnerable to the effect of glutamate, and after eight days in culture they reach their full maturation and die after glutamate excitotoxic insult (Ankarcrona et al. 1995; Contestabile 2002). Our data highlight Lamin A/C as a crucial protein in neural maturation. Across three different models, we consistently observed that *LMNA* gene knock-down rendered neuronal cells unresponsive to glutamate. These findings align with previous evidence suggesting that neuronal stem cells not only protect themselves from excitotoxic glutamate damage but also utilize this neurotransmitter to promote proliferation via an autocrine mechanism (Teng et al. 2023; Lladó et al. 2004).

A decrease in the expression of chemokines and cytokines may prevent the initiation of neuroinflammatory processes that lead neuronal cells to necrosis (Ramesh et al. 2013). The relationship between Lamin A/C expression and inflammation is not well understood yet. More robust evidence has been proposed around the role of mutations in the *LMNA* gene in overactive or dysregulated myocardial inflammatory responses in Lamin A/C cardiomyopathies (Gerbino et al. 2021). Kim et al. observed that there might be a link between Lamin A/C over-expression and the activation of pro-inflammatory pathways in macrophages (Kim et al. 2018). Elevated Lamin A/C levels are also associated with ERK1/2 signalling during T lymphocyte activation (Gonzalez-Granado et al. 2014). In our study, we observed a correlation between a decrease in Lamin A/C expression and the down-regulation of *Cxcl10*, *Ccl5* and *Ccl7* gene expression levels from microarray profiling. Although with some expected differences due to the different species and models utilized, our in vitro observations from rat cerebellar GCs and SH-SY5Y human neuronal cells were also confirmed in a *Lmna*-KO in vivo mouse model. The diminished concentration of intracellular calcium in *LMNA*-KD GCs and SH-SY5Y raises the possibility of an interesting scenario, where the reduction in cytokines and chemokines expression in our models is a direct consequence of reduced calcium influx observed in our experiments. Several groups have demonstrated how calcium signalling and cytokine production are interlinked and are key drivers for cell death in neurons and other cell types (Eskiocak et al. 2023; Ramadan et al. 2011; Lilienbaum and Israël, 2003).

The crucial role of the nuclear membrane may be behind our results on the involvement of the *LMNA* gene in calcium signalling and protection from neurotoxicity. Nuclear lamins, including Lamin A/C, are indeed essential structural proteins for maintaining nuclear morphology, integrity and permeability (Dahl and Klinowski 2011; Philip and Dahl 2008). Numerous studies have demonstrated a correlation between the alteration of the nuclear membrane structure and cell death mechanisms induced by calcium influx in response to glutamate (Bano et al. 2010a; Bano et al., 2010b). In particular, the research of Bano and colleagues has explored the hierarchical sequence of events triggered by glutamate neurotoxicity, highlighting how alterations in the permeability of the nuclear membrane represent the "point of no return" in the neurotoxic process. Lamin A/C plays an important role in the differentiation of osteoblasts, adipocytes and mesenchymal stem cells by regulating certain elements of Wnt/β-catenin and Notch signalling pathways (Bermeo et al. 2015; Scaffidi and Misteli 2008), and these interconnected pathways (Walsh and Andrews 2003) are functional in the maturation process of the cerebellum (Deutschmann et al. 2011; Saito and Takeshima 2006). Additionally, Wnt/β-catenin signalling in neural progenitor cells has been shown to promote neuroinflammation (Sebo et al. 2025). This aligns with our findings that the silencing or deletion of the *LMNA* gene leads to a significant reduction in the expression of inflammation-related genes as compared to the wild-type condition.

## Conclusions

**Fig. 8:**
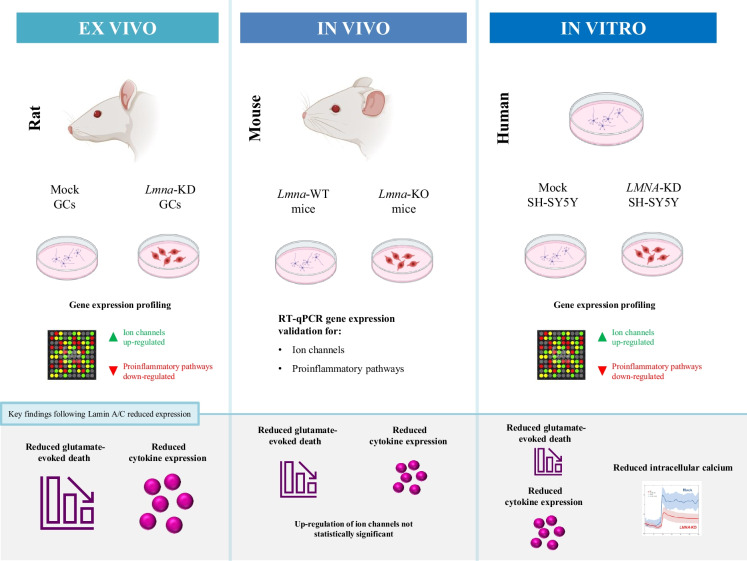
Graphical summary of the results shown in this paper

Our study demonstrated that Lamin A/C influences the maturation of rat cerebellar GCs and is necessary for the intracellular calcium influxes. In addition, Lamin A/C regulates the expression of pro-inflammatory cytokine pathways and its down-regulation protects neurons from glutamate-evoked excitotoxicity, as we summarized in the graphical abstract (Fig. 8). It is beyond the scope of this manuscript to understand how Lamin A/C regulates the expression of the genes that belong to the pathways described above.

## Supplementary Information

Below is the link to the electronic supplementary material.

Supplementary file1 (XLSX 18 KB)

Supplementary file2 (DOCX 178 KB)

## Data Availability

The microarray gene expression data related to rat GCs are publicly available from the Gene Expression Omnibus database (https://www.ncbi.nlm.nih.gov/geo), under the Series GSE247980. The microarray data of SH-SY5Y cells were already deposited in GEO repository, under the Series GSE30677.
