# Supplementary material for: Lamin A/C regulates cerebellar granule cell maturation
Source: Cell Biol Toxicol. 2025 Apr 5;41(1):66. doi: 10.1007/s10565-025-10011-z (PMC11972193; doi:10.1007/s10565-025-10011-z)
Supplement: Supplementary file 2 — Supplementary file2 (DOCX 178 KB) [file 10565_2025_10011_MOESM2_ESM.docx]

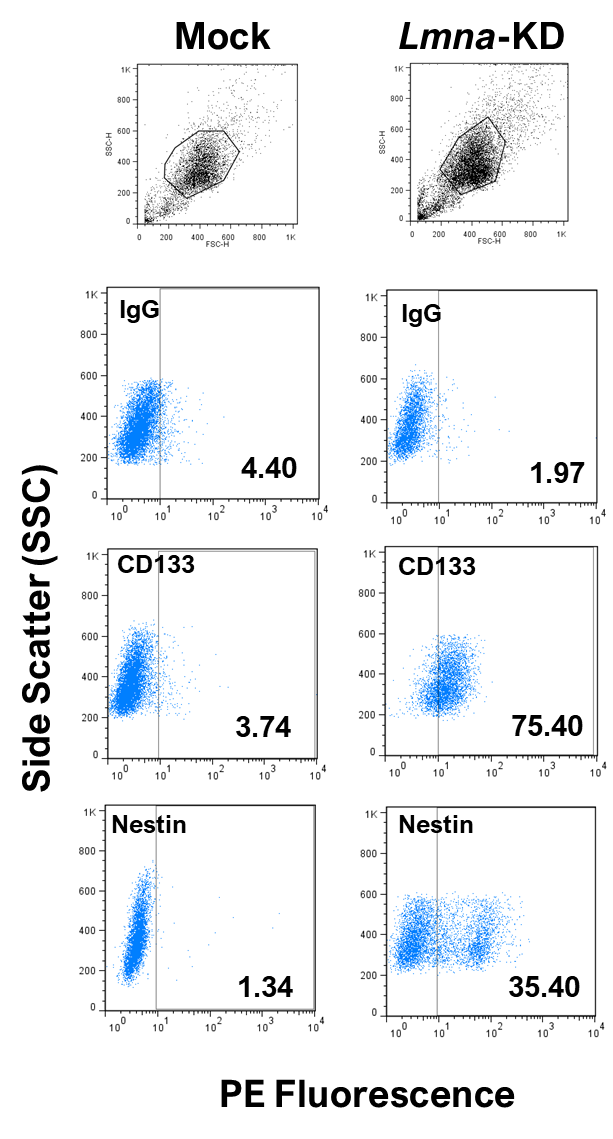


**Supplementary Fig. 1.** FACS analysis of the expression of CD133 and Nestin protein in rat GCs. Mock and *Lmna*-KD GCs at 8DIV culture were stained with either PE Mouse IgG1 (κ Isotype Control, clone MOPC-21; BD-Pharmingen) or PE Mouse Anti-Human CD133 antibody (clone W6B3C1; BD-Pharmingen). For Nestin, cells were fixed (BD Cytofix™ buffer) for 20 min at RT, permeabilized with BD Phosflow™ Perm Buffer I, following the manufacturer’s protocol. The samples were then stained with PE Mouse anti-Nestin (Clone 25/NESTIN; BD-Pharmingen). The samples were then washed with PBS and measured by a FACSCalibur cytometer (Becton Dickinson) and CellQuest Pro BD software (Becton Dickinson). Flow cytometry data were analyzed by FlowJo^TM^ data analysis platform v.8.0 (Becton Dickinson). Aggregates and debris were excluded from the analysis gating on the scatter cytograms (FSC vs SSC) of the cell population, characteristics of viable cells. The number present in the cytograms represents the percentage of positive cells in each condition, based on the region identified in the control samples (IgG) to define a background.
